# Inhibition of myotube formation by platelet-derived growth factor subunit B in QM7 cells

**DOI:** 10.5713/ab.24.0262

**Published:** 2024-08-23

**Authors:** Sarang Choi, Sangsu Shin

**Affiliations:** 1 Department of Animal Science and Biotechnology, Kyungpook National University, Sangju 37224, Korea; 2 Research Institute for Innovative Animal Science, Kyungpook National University, Sangju 37224, Korea

**Keywords:** Muscle Differentiation, Myoblast, Myogenesis, Platelet-derived Growth Factor Subunit B, QM7 Cell

## Abstract

**Objective:**

The primary objective of this study was to investigate the role and regulatory mechanisms of platelet-derived growth factor subunit B (*PDGFB*) in muscle differentiation.

**Methods:**

In this study, a vector for *PDGFB* was designed and transfected into quail muscle cells to investigate its role and regulatory mechanism during muscle formation. To investigate the inhibitory mechanisms of *PDGFB* on myogenic differentiation, the mRNA expression levels of various genes and the phosphorylation of extracellular signal-regulated kinase 1/2 (ERK 1/2), both known to regulate muscle development and differentiation were compared.

**Results:**

*PDGFB*-overexpressed (OE) cells formed morphologically shorter and thinner myotubes and demonstrated a smaller total myotube area than did the control cells. This result was also confirmed at the molecular level by a reduced amount of myosin heavy chain protein in the *PDGFB*-OE cells. Therefore, *PDGFB* inhibits the differentiation of muscle cells. Additionally, the expression of myogenin (*MYOG*) significantly decreased in the *PDGFB*-OE cells on days 2 and 4 compared with that in the control cells. The phosphorylation of ERK 1/2, an upstream protein that inhibits *MYOG* expression, increased in the *PDGFB*-OE cells on day 4 compared with that in the control cells. The decreased expression of *MYOG* in the *PDGFB*-OE cells increased by inhibition ERK 1/2 phosphorylation.

**Conclusion:**

*PDGFB* may suppress myogenesis by reducing *MYOG* expression through ERK 1/2 phosphorylation. These findings can help understand muscle differentiation and potentially improve poultry meat production.

## INTRODUCTION

The production and consumption of poultry meat are increasing worldwide [1]. Currently, there have been significant advancements in chicken growth, feed efficiency, and breast muscle size, resulting in chickens weighing considerably more than they did in the past [2]. However, the rapid growth of muscles has been reported to have adverse effects on poultry meat quality, such as poor cohesiveness of meat due to immaturity of intramuscular connective tissue and pectoral muscle disease [3]. It also leads to reduced water holding capacity after death [4]. Hence, studies have been conducted to control these effects and enhance both production rates and quality. A comprehensive understanding of muscle proliferation and differentiation may hold the key to enhancing poultry growth. Therefore, several efforts have been made to enhance the economic value of poultry production, particularly by exploring gene expression and pathways related to muscle cell proliferation and differentiation.

The skeletal muscle, a fundamental tissue composed of bundles of muscle fibers, plays a crucial role in movement, support, and overall physiological functions in the body. Muscle formation is a complex process involving the fusion of myogenic precursor cells called myoblasts. This fusion is achieved through various gene expressions and regulation processes. Generally, muscle development and differentiation are regulated by paired box (*PAX*) genes and myogenic regulatory factors (MRFs) [5]. *PAX* genes, such as *PAX3* and *PAX7*, function as transcription factors and regulate the fate of proliferation and differentiation during early-stage myogenic development [6]. MRFs, including myogenic factor 5 (*MYF5*), myogenic differentiation 1 (*MYOD1*), myogenin (*MYOG*), and myogenic factor 6 (*MYF6*), induce myogenic differentiation in cells [7].

Platelet-derived growth factor subunit B (PDGFB), which is a secretory protein expressed in skeletal muscles, is a signaling molecule involved in critical cellular processes, including proliferation and development [8–10]. The *PDGFB* gene is expressed in cases of tissue damage and inflammation, thereby contributing to skeletal muscle remodeling and recovery [11,12]. *PDGFB* is emerging as a major component in the production process of cultured meat because it affects various cells and promotes myoblast proliferation [12,13]. Previous studies have reported that *PDGFB* stimulates myoblast proliferation but inhibits myoblast fusion and reduces creatine phosphokinase activity [14–16]. A recent study showed that PDGF-BB activates myoblast proliferation and enhances muscle regeneration in patients with Duchenne muscle dystrophy [11]. While the role of *PDGFB* in muscles has been studied extensively, information regarding its detailed mechanism in skeletal muscles and in particular, its impact on the expression of *PAX* genes and MRFs, which are critical regulators of skeletal muscle differentiation, is lacking. The primary objective of this study was to investigate the effect of *PDGFB* on muscle differentiation in quail muscle (quail muscle clone 7, QM7) cells and elucidate its regulatory role in the expression of *PAX* genes and MRFs. In this study, QM7 cells were transfected with a *PDGFB* expression vector, and muscle differentiation and gene expression patterns were investigated in *PDGFB*-OE cells during the 4-day differentiation process.

## MATERIALS AND METHODS

### *PDGFB* gene cloning and expression vector construction

The coding sequence of quail *PDGFB* (GenBank accession no.: XM_015860305.2) was amplified by polymerase chain reaction (PCR) with a primer set of PDGFB-forward and PDGFB-reverse and then cloned into the pGEM-T easy vector (Promega, Madison, WI, USA). The sequence of the cloned quail *PDGFB* was confirmed by sequencing. To construct the expression vector, the hemagglutinin (HA)-tag sequence was added just before the stop codon of the *PDGFB* gene by PCR, employing PDGFB-forward and PDGFB-HA-reverse primers. Finally, the amplified sequence was inserted into the multicloning site of the pcDNA3.1 expression vector (Invitrogen, Grand Island, NY, USA). The primer sequences are shown in Table 1.

### Cell culture and transfection of cells

QM7 cells (American Type Culture Collection, Manassas, VA, USA) were cultured in a growth medium, namely Medium 199 containing 10% fetal bovine serum (FBS; Gibco, Grand Island, NY, USA), 1% chicken serum (CS; Sigma-Aldrich, St. Louis, MO, USA), and 1% antibiotic-antimycotic (ABAM; Gibco, Waltham, MA, USA). The cells were cultured in an incubator at 37°C under 5% CO_2_ conditions. They were subcultured to prevent differentiation before filling the dishes. To induce differentiation, the first medium was replaced with differentiation medium, i.e., Medium 199 containing 0.5% FBS, 0.1% CS, and 1% ABAM. Fourteen 35-mm dishes were seeded with 6×10^5^ cells 18 hours before transfection. *PDGFB*-containing or empty expression vectors were transfected 22 hours before the initiation of differentiation using jetOPTIMUS DNA transfection reagent (Polyplus, Illkirch, France) according to the manufacturer’s protocols. The differentiation medium was replaced with fresh medium every 2 days.

### Cell sample collection

Differentiation was monitored at the following four distinct time points: day 0, marking the onset of differentiation; day 1, indicating the shift in myogenic gene expression; day 2, signifying the active formation of myotubes; and day 4, denoting sufficient myotube formation. mRNA samples were collected at all four time points (days 0, 1, 2, and 4) or on day 4; protein samples, at two time points (days 0 and 4); and immunofluorescence samples, exclusively on day 4. A sample was meticulously prepared at each designated time point for subsequent analysis.

For inhibitor-treated cells, differentiation medium containing PD98059 (Cell Signaling Technology, Danvers, MA, USA) was added on days 0 and 2. Protein and mRNA samples from the inhibitor-treated cells were collected on days 2 and 4, respectively.

### Immunofluorescence staining of cells and myotube area analysis

For myotube staining, the cells were initially washed with phosphate-buffered saline (PBS) and then fixed with 10% neutral formalin for 15 minutes. Subsequently, the cells were permeabilized using 0.3% NP40 for 20 minutes and blocked with 5% non-fat dry milk in PBS containing 0.1% Tween-20 (PBST) for 30 minutes. The cells were then stained with the primary antibody for 1 hour and washed three times using PBST. Next, the cells were treated with the secondary antibody. During the antibody treatment, the concentration of skim milk was reduced to 1%. The cell nuclei were counterstained with 4′,6-diamidino-2-phenylindole (DAPI) for 5 minutes, and the cells were washed three times with PBST. The images of the stained cells were captured using an inverted fluorescence microscope (CKX53; Olympus, Tokyo, Japan). The area of the myotubes was measured in the myosin heavy chain (MyHC)-positive cells.

### Antibodies and chemicals

Anti-MyHC MF20 (Developmental Studies Hybridoma Bank, Iowa City, IA, USA) and anti-MyHC NA4 (Developmental Studies Hybridoma Bank, USA) were used for immunofluorescence staining and western blotting, respectively. For immunofluorescence staining, anti-mouse immunoglobulin G (IgG) conjugated with CruzFluor594 (Santa Cruz Biotechnology, Dallas, TX, USA) was used as the secondary antibody. Anti-extracellular signal-regulated kinase 1/2 (ERK 1/2) and anti-phospho ERK were obtained from Cell Signaling Technology (USA). Anti-HA-tag antibody was procured from Santa Cruz Biotechnology (USA). The secondary antibodies, namely goat-IgG mouse-horseradish peroxidase (HRP) and goat-IgG rabbit-HRP were obtained from Thermo Fisher Scientific (Carlsbad, CA, USA). PD98059 was purchased from Cell Signaling Technology (USA).

### Western blotting

The cells were washed with PBS, and the proteins were extracted using a 1× lysis buffer. An equal volume of 2× Laemmli sample buffer (BioRad, Hercules, CA, USA) containing β-mercaptoethanol was mixed with the extracted proteins. The mixture was boiled for 5 minutes at 100°C, and Coomassie staining was performed for protein quantification. The protein samples were separated by polyacrylamide gel electrophoresis and transferred onto a polyvinylidene fluoride membrane. Subsequently, the non-specific antigens were blocked using 5% non-fat dry milk or bovine serum albumin (BSA) (Biosesang, Yongin, Korea) in Tris-buffered saline mixed with Tween-20 (TBST) for 1 hour. After incubating with the primary antibodies overnight at 4°C, the secondary antibodies were stored at room temperature for 1 hour. During treatment with antibodies, the concentration of skim milk or BSA was lowered to 2.5%. After processing the enhanced chemiluminescence (ECL) solution, images were detected using the Amersham ImageQuant 500 (Cytiva, Marlborough, MA, USA).

### RNA isolation and cDNA synthesis

Total mRNA was extracted from the samples obtained on days 0, 1, 2, and 4 of differentiation using RNAiso plus (Takara Bio Inc., Shiga, Japan). The qualities of isolated RNA were confirmed using the P200 Micro-volume spectrophotometer (Biosis Design, Gwangmyeong, Korea) and electrophoresis. Using 1 μg RNA, cDNA synthesis was performed using the DiaStar RT kit (SolGent, Daejeon, Korea) according to the manufacturer’s instructions.

### Quantitative real-time polymerase chain reaction

Quantitative real-time PCR (qRT-PCR) was performed using the Bio Rad CFX Connect Real-Time PCR Detection System (Bio Rad, Hercules, CA, USA). Table 1 shows the primer pairs designed for qRT-PCR. The single amplicon of each primer set was confirmed by a melting curve. Target genes were normalized to glyceraldehyde-3-phosphate dehydrogenase (GAPDH). The expression levels of the target genes were calculated using the 2^−ΔΔCt^ method.

### Statistical analysis

All experiments were independently conducted at least three times. The data were presented as mean±standard error of the mean (SEM) and analyzed using the Student’s *t*-test and two-way analysis of variance (ANOVA) in the R package (R Foundation for Statistical Computing, Vienna, Austria). For multiple groups showing significance in the two-way ANOVA test, pos-hoc comparisons were conducted using the Duncan’s multiple range test. Statistical significance was defined as p<0.05.

## RESULTS

### Inhibitory effect of myotube formation by *PDGFB* overexpression

**Figure 1:**
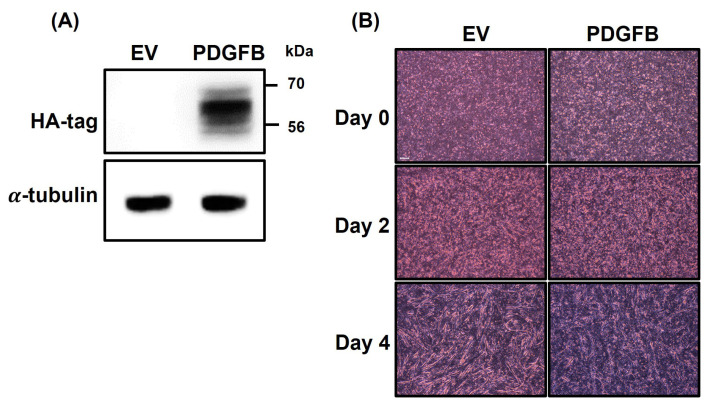
The overexpression of HA-labeled PDGFB and differentiation of QM7 cells. (A) Overexpression of *PDGFB* is confirmed by western blot analysis using HA-tag antibodies on day 0. (B) Myotube formation of EV and *PDGFB*-OE cells is analyzed on days 0, 2, and 4. Scale bar, 200 μm. HA, hemagglutinin; PDGFB, platelet-derived growth factor subunit B; EV, empty vector; OE, overexpressed.

To examine the effect of *PDGFB* on muscle differentiation, an overexpression vector was designed and transfected into QM7 cells. Subsequently, the differentiation process was conducted over a 4-day period (Figure 1). First, successful transfection of cells with HA-tagged *PDGFB* was confirmed (Figure 1A). Western blot analysis using HA-tag antibodies on day 0 of differentiation revealed that compared with the empty vector-transfected (EV) cells, the *PDGFB*-OE cells showed a distinct HA-tag expression. This result confirms the effective overexpression of *PDGFB* in the QM7 cells. On day 0, no significant differences were observed between the groups because the myotubes had not yet formed (Figure 1B). However, the *PDGFB*-OE cells exhibited shorter and thinner myotubes than did the EV cells on day 2. These differences in myotube formation between the groups became more noticeable on day 4. The *PDGFB*-OE cells did not form long, well-developed myotubes like the EV cells did.

**Figure 2:**
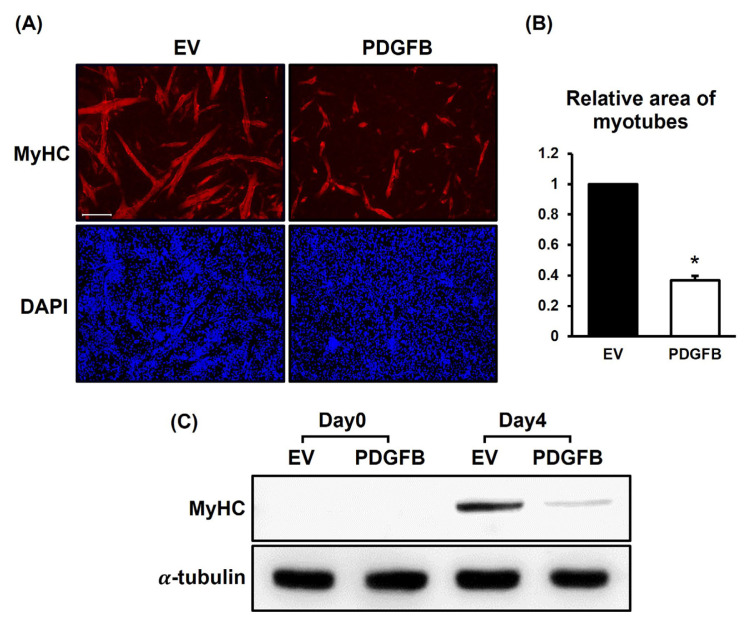
Stained myotubes, measurement of myotube area, and protein expression level of MyHC in EV and *PDGFB*-OE cells. (A) Myotubes (red) and nuclei (blue) are stained with MyHC antibodies and DAPI, respectively on day 4 of differentiation. (B) The relative area of stained myotubes in *PDGFB*-OE cells is measured compared with the EV cells. (C) Expression levels of MyHC are analyzed by western blot on day 0 (initiation of differentiation) and day 4 (terminal differentiation). Scale bar, 200 μm. MyHC, myosin heavy chain; EV, empty vector; PDGFB, platelet-derived growth factor subunit B; OE, overexpressed; DAPI, 4′,6-diamidino-2-phenylindole. * p<0.001.

Immunostaining of myotubes with MyHC antibodies and DAPI was conducted to analyze the area of myotubes on day 4 (Figure 2A and 2B). The myotubes of the *PDGFB*-OE cells were poorly formed; moreover, they had more mononucleated cells than did the control cells (Figure 2A). These results were consistent with those observed in Figure 1B. The *PDGFB*-OE cells had ≤40% of the total myotube area than did the EV cells (p<0.001) (Figure 2B). Additionally, to confirm the degree of differentiation between the groups at the protein level after *PDGFB* overexpression, western blotting analysis of the samples was conducted on days 0 and 4 using an antibody targeting MyHC (Figure 2C). In both the groups, MyHC protein was not detected on day 0, whereas it was detected on day 4. The MyHC protein on day 4 was remarkably decreased in the *PDGFB*-OE cells. These results indicated that *PDGFB* negatively affects myotube formation in QM7 cells, resulting in reduced myotube formation and subsequent decreased expression of MyHC protein.

### Regulation of MRF expressions by *PDGFB* during muscle differentiation

**Figure 3:**
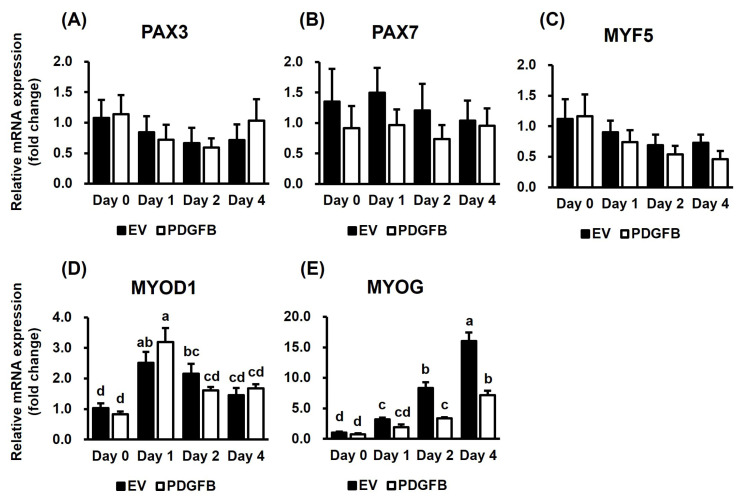
Analysis of gene affecting myogenesis by *PDGFB* overexpression. (A-E) The expression of *PAX3*, *PAX7*, *MYF5*, *MYOD1*, and *MYOG* is analyzed by qRT-PCR between the two groups. *PDGFB*, platelet-derived growth factor subunit B; *PAX*, paired box; *MYF5*, myogenic factor 5; *MYOD1*, myogenic differentiation 1; *MYOG*, myogenin; EV, empty vector; qRT-PCR, quantitative real-time polymerase chain reaction. Columns marked with different letters indicate significant differences at p<0.001, while columns marked with the same letters indicate no significant differences.

The expression of *PAX* and *MRF* genes due to *PDGFB* overexpression during myogenesis was analyzed by qRT-PCR (Figure 3). Though the expression of *PAX3* and *PAX7* was not significantly different between the groups (Figure 3A and 3B), *PAX7* expression reduced in the *PDGFB*-OE during differentiation. Subsequently, the expression of MRFs was examined. The expression of *MYF5* did not differ between the groups (Figure 3C). While the expression of *MYOD1* showed no difference between the groups, it changed significantly during differentiation in both the groups (p<0.001) (Figure 3D).

The expression of *MYOG* significantly increased during myogenesis in both the groups (p<0.001) (Figure 3E). However, its expression decreased in the *PDGFB*-OE cells when compared with that in the EV cells on days 2 and day 4 (p< 0.001). The differentiation day and treatment interaction significantly influenced *MYOG* expression (p<0.001). These data indicated that *PDGFB* interferes with *MYOG* expression among MRFs, leading to incorrect myotube formation.

### Increased phosphorylated ERK 1/2 by *PDGFB* overexpression during myogenic differentiation

**Figure 4:**
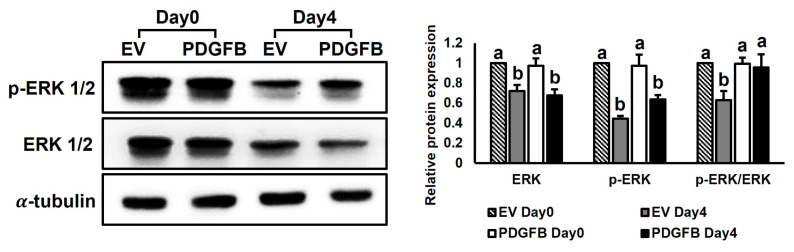
Analysis of ERK 1/2 phosphorylation by *PDGFB* overexpression. The expression level of total ERK 1/2 and ERK 1/2 phosphorylation are measured by western blot on day 0 and day 4 in both groups. ERK 1/2, extracellular signal-regulated kinase 1/2; *PDGFB*, platelet-derived growth factor subunit B; OE, overexpressed; EV, empty vector. Columns labeled with different letters show significant differences at p<0.05, while columns labeled with the same letters show no significant differences.

To investigate the signaling pathway altered by *PDGFB* overexpression, ERK 1/2, which is known to regulate cell proliferation and differentiation, was analyzed using western blotting (Figure 4). The total ERK 1/2 expression decreased in both the groups during differentiation (p<0.001), but it decreased more in the *PDGFB*-OE cells than in the EV cells on day 4. ERK 1/2 phosphorylation also decreased on day 4 when compared with that on day 0 in both the groups (p<0.001). However, this phosphorylation was reduced to a lesser extent in the *PDGFB*-OE cells than that in the EV cells on day 4 (p<0.05). These results suggested that ERK phosphorylation decreases during differentiation, and *PDGFB* may interfere with this signaling pathway.

### Increased *MYOG* expression in *PDGFB*-OE cells by ERK 1/2 inhibitor

**Figure 5:**
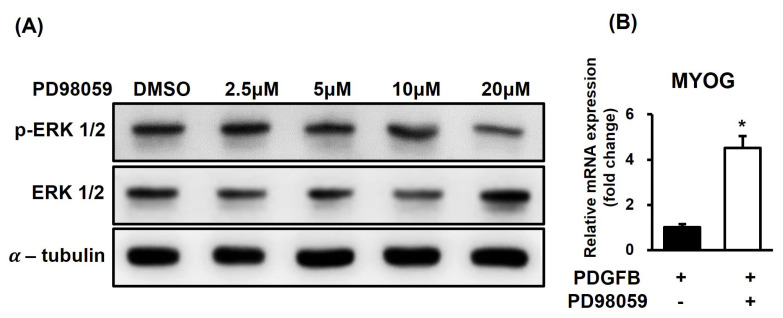
Effect of PD98059 on ERK 1/2 phosphorylation and expression of *MYOG* in QM7 cells. (A) *PDGFB*-OE cells were treated with 2.5, 5, 10, and 20 μM PD98059 on day 0. On day 2, the expression level of ERK 1/2 phosphorylation is analyzed by western blot. (B) *PDGFB*-OE cells were treated with 20 μM PD98059 on days 0 and 2 followed by changing fresh medium. Expression of MYOG in *PDGFB*-OE cells is confirmed with qRT-PCR compared with the EV cells on day 4. ERK 1/2, extracellular signal-regulated kinase 1/2; MYOG, myogenin; *PDGFB*, platelet-derived growth factor subunit B; OE, overexpressed; DMSO, dimethyl sulfoxide; qRT-PCR, quantitative real-time polymerase chain reaction. * p<0.01.

To investigate the effect of ERK 1/2 inhibition on *MYOG* expression in the *PDGFB*-cells, the concentration of inhibitor of ERK 1/2 was determined in QM7 cells, and *MYOG* expression was examined (Figure 5). The inhibitor concentration was determined based on previous studies [17,18] according to the recommendations of the manufacturer (Figure 5A). ERK 1/2 phosphorylation was effectively inhibited at a concentration of 20 μM, which was used in subsequent experiments. Concentrations above 20 μM resulted in cell death and were therefore not utilized. Subsequently, when ERK 1/2 phosphorylation in the *PDGFB*-OE cells was inhibited, the expression of *MYOG* increased when compared with that in the controls on day 4 (p<0.01) (Figure 5B). This study demonstrated that treatment of these inhibitors, *PDGFB* reduces the expression of *MYOG* through the ERK phosphorylation pathway.

## DISCUSSION

In this study, the overexpression of *PDGFB* led to a significant inhibition of myotube formation in QM7 cells, which was evident by the formation of shorter and thinner myotubes and reduction in the total myotube area than in the control cells. These findings are consistent with those of a previous study that reported a decreased frequency of myosin-positive cells and suppression of biochemical differentiation [12,16]. Furthermore, Jin et al [15] reported that *PDGFB* inhibits the expression of creatine phosphokinase and MyHC. We also detected a reduction in the expression of MyHC, a marker of differentiated muscle cells [19], in multinucleated myotubes, which conforms to the results of a previous study [20]. In this study, the decreased protein level of MyHC was due to diminished myotube formation in the *PDGFB*-OE cells when compared with that in the control cells. Collectively, these results support the notion that *PDGFB* acts as a negative regulator of muscle differentiation in QM7 cells.

This study aimed to elucidate the correlations and molecular mechanisms between *PDGFB* and muscle-related genes and the effects on muscle development and differentiation. Initially, *PAX3* and *PAX7* serve as markers for myogenic progenitor cells and guide their initiation into skeletal muscle differentiation [6]. In this study, *PAX* genes did not show significant differences between the groups, suggesting that *PDGFB* does not affect early muscle cell proliferation and development.

Muscle-specific basic helix-loop-helix transcription factors, known as MRFs, are capable of inducing myogenic differentiation in cells [21]. Furthermore, the expression of MRFs follows a unique and sequential pattern while regulating myogenesis [22,23]. In this study, the expression of *MYF5* and *MYOD1* also did not differ between the groups. These genes regulate early myogenic differentiation, and their deficiency delays myogenesis, leading to death immediately after birth [24,25]. Although complete myogenesis was not achieved in our study, the incomplete formation of myotubes in the *PDGFB*-OE cells suggests that *PDGFB* does not affect the early stages of differentiations, as evidenced by the unchanged *MYOD1* expression levels.

Compared with the EV cells, the *PDGFB*-OE cells demonstrated a significant reduction in the expression of *MYOG*. *MYOG* expression is induced by *MYOD1*, which in turn induces differentiation leading to terminal fusion into myotubes [26,27]. In *MYOG*-knockout mouse models, a defect in *MYOG* results in severe reduction of all skeletal muscles [28]. Moreover, the frequency of MYOG-(+) cells is reduced in cultures exposed to PDGF-BB, suggesting that PDGF-BB may delay *MYOG* expression [29]. Therefore, the reduced expression of *MYOG* in the *PDGFB*-OE cells suggests that *PDGFB* interferes with *MYOG* expression in QM7 cells, leading to inhibition of muscle differentiation.

PDGFB functions in an autocrine/paracrine manner in both myoblasts and myotubes [14]. The PDGF family includes various subunits (A, B, C, and D). They can be secreted as homodimers, such as PDGF-AA, PDGF-BB, PDGF-CC, and PDGF-DD, or heterodimers, such as PDGF-AB [9,30]. PDGF released into the extracellular space binds to PDGF receptors (PDGFR), including PDGFRαα, PDGFRαβ, and PDGFRββ, on the cell surface and initiates a cascade of cellular responses [31]. Specifically, PDGF-BB binds to all the PDGFR forms, whereas PDGF-AB selectively binds to PDGFR-αα and PDGFR-αβ [8,32]. The binding of PDGFB with PDGFR triggers various downstream pathways, including the Ras/mitogen-activated protein kinase (MAPK) and ERK pathways [33–35]. Furthermore, the Ras-activated ERK (Ras-ERK) pathway, a downstream effector kinase of PDGFB-mediated PDGFR activation, plays a crucial role in cellular functions [36,37].

To explore the relationship between ERK 1/2 and inhibition of muscle differentiation by *PDGFB*, ERK 1/2 phosphorylation was analyzed. While ERK 1/2 expression and ERK 1/2 phosphorylation both decreased during differentiation, ERK 1/2 phosphorylation was found to be higher in the *PDGFB*-OE cells than that in the EV cells, indicating its involvement in the regulation of myoblast differentiation. Although ERK 1/2 promotes myoblast proliferation and affects cell-cycle regulation, it is not essential for muscle gene expression or cell fusion [38]. Our study results suggest that under normal circumstances, ERK 1/2 phosphorylation should decrease during muscle differentiation, but *PDGFB* overexpression increases ERK 1/2 activation. To better identify the aforementioned pathway in the *PDGFB*-OE cells, PD98059, an inhibitor of ERK 1/2 phosphorylation, was used. *MYOG* expression higher in the *PDGFB*-OE cells than that in the controls when ERK 1/2 phosphorylation was inhibited. This finding was consistent with that of a previous study, which showed that ERK 1/2 inhibition upregulates *MYOG* expression and promotes myoblast differentiation [39]. The fraction of MYOG-(+) nuclei significantly increased in the differentiation medium supplemented with an ERK 1/2 inhibitor compared with that in the differentiation medium alone. Recently, Takahashi et al [40] clarified the induction of skeletal myocyte differentiation using DA-Raf, an antagonist of the Ras-ERK pathway. Reduction in DA-Raf was found to decrease the expression of *MYOG* and increase ERK 1/2 phosphorylation. Therefore, this study surmised that *PDGFB* activates ERK 1/2 phosphorylation and ERK 1/2 activation inhibits *MYOG* expression.

In conclusion, *PDGFB* overexpression inhibited myotube formation and had a negative impact on muscle differentiation in QM7 cells. Despite no significant effects on the early stages of differentiation, it significantly reduced the expression of *MYOG*, a key regulator of terminal muscle differentiation, in the *PDGFB*-OE cells. Furthermore, this study also demonstrated that *PDGFB* activated ERK 1/2 phosphorylation, which may inhibit *MYOG* expression, thus providing insights into its role as a negative regulator of muscle differentiation. Further study is necessary to better understand the signaling pathway involved in the reduction of *MYOG* by *PDGFB* during myogenesis.

## Figures and Tables

**Table 1:** List of primers used for cloning and real-time polymerase chain reaction

Primers		Sequences 5′→3′	Annealing temperature (°C)	Reference genes (GenBank no.)
Cloning	PDGFB-F	5′-CAG CTG CCA GTG CTT CCC-3′	59.0	XM_015860305.2
	PDGFB-R	5′-TGC TCT CTC TCC CTT GCC AG-3′		
	PDGFB-HA-R	5′- TCA CAG GCT AGC GTA ATC TGG CAC ATC GTA TGG GTA TGC TAT GAG GAT TTC TTT CAG-3′		
qPCR	GAPDH-F	5′ - GAG GGT AGT GAA GGC TGC TG - 3′	58.0	XM_015873412.2
	GAPDH-R	5′ - ACC AGG AAA CAA GCT TGA CG - 3′		
	PAX3-F	5′ - AGC AAC TGG AAG AGC TGG AAA G -3′	64.0	XM_015871337.2
	PAX3-R	5′ - CTC CTG GGA TCA GGT GGT TAA A - 3′		
	PAX7-F	5′ - AGC TGG CAG AGA TGG AGT TG - 3′	64.0	XM_032448793.1
	PAX7-R	5′ - CTA GTG GTG GTG GTG GCA AA - 3′		
	MYF5-F	5′ - AGG AGG CTG AAG AAA GTG AAC C -3′	60.0	XM_015857363.2
	MYF5-R	5′ - TAG TTC TCC ACC TGT TCC CTC A - 3′		
	MYOD1-F	5′ - CAG CTA CTA CAC AGA ATC ACC AA - 3′	63.2	XM_015864142.1
	MYOD1-R	5′ - TCC CTT CAG CAA CAG CTT CA - 3′		
	MYOG-F	5′ - TGC CCA AGG TGG AGA TCC TA - 3′	63.2	XM_015884883.2
	MYOG-R	5′ - GGG TTG GTG CCA AAC TCC AG - 3′		

F, forward; R, reverse; PDGFB, platelet-derived growth factor subunit B; HA, hemagglutinin; GAPDH, glyceraldehyde-3-phosphate dehydrogenase; PAX, paired box; MYF5, myogenic factor 5; MYOD1, myogenic differentiation 1; MYOG, myogenin.
